# Changes in the Epidemiological Features of Influenza After the COVID-19 Pandemic in China, the United States, and Australia: Updated Surveillance Data for Influenza Activity

**DOI:** 10.2196/47370

**Published:** 2024-10-09

**Authors:** Mingyue Jiang, Mengmeng Jia, Qing Wang, Yanxia Sun, Yunshao Xu, Peixi Dai, Weizhong Yang, Luzhao Feng

**Affiliations:** 1 School of Population Medicine and Public Health Chinese Academy of Medical Sciences & Peking Union Medical College Beijing China; 2 National Institute of Pathogen Biology Chinese Academy of Medical Sciences & Peking Union Medical College Beijing China; 3 Chinese Center for Disease Control and Prevention Beijing China

**Keywords:** influenza, seasonal variation, COVID-19 pandemic, stringency index

## Abstract

**Background:**

There has been a global decrease in seasonal influenza activity since the onset of the COVID-19 pandemic.

**Objective:**

We aimed to describe influenza activity during the 2021/2022 season and compare it to the trends from 2012 to 2023. We also explored the influence of social and public health prevention measures during the COVID-19 pandemic on influenza activity.

**Methods:**

We obtained influenza data from January 1, 2012, to February 5, 2023, from publicly available platforms for China, the United States, and Australia. Mitigation measures were evaluated per the stringency index, a composite index with 9 measures. A general additive model was used to assess the stringency index and the influenza positivity rate correlation, and the deviance explained was calculated.

**Results:**

We used over 200,000 influenza surveillance data. Influenza activity remained low in the United States and Australia during the 2021/2022 season. However, it increased in the United States with a positive rate of 26.2% in the 49th week of 2022. During the 2021/2022 season, influenza activity significantly increased compared with the previous year in southern and northern China, with peak positivity rates of 28.1% and 35.1% in the second week of 2022, respectively. After the COVID-19 pandemic, the dominant influenza virus genotype in China was type B/Victoria, during the 2021/2022 season, and accounted for >98% (24,541/24,908 in the South and 20,543/20,634 in the North) of all cases. Influenza virus type B/Yamagata was not detected in all these areas after the COVID-19 pandemic. Several measures individually significantly influence local influenza activity, except for influenza type B in Australia. When combined with all the measures, the deviance explained values for influenza A and B were 87.4% (*P*<.05 for measures of close public transport and restrictions on international travel) and 77.6% in southern China and 83.4% (*P*<.05 for measures of school closing and close public transport) and 81.4% in northern China, respectively. In the United States, the association was relatively stronger, with deviance-explained values of 98.6% for influenza A and 99.1% (*P*<.05 for measures of restrictions on international travel and public information campaign) for influenza B. There were no discernible effects on influenza B activity in Australia between 2020 and 2022 due to the incredibly low positive rate of influenza B. Additionally, the deviance explained values were 95.8% (*P*<.05 for measures of restrictions on gathering size and restrictions on international travel) for influenza A and 72.7% for influenza B.

**Conclusions:**

Influenza activity has increased gradually since 2021. Mitigation measures for COVID-19 showed correlations with influenza activity, mainly driven by the early stage of the pandemic. During late 2021 and 2022, the influence of mitigation management for COVID-19 seemingly decreased gradually, as the activity of influenza increased compared to the 2020/2021 season.

## Introduction

Seasonal influenza is an epidemic disease caused by the influenza virus with a high burden and severity. The World Health Organization (WHO) and the Centers for Disease Control and Prevention of most countries jointly coordinate influenza surveillance and report weekly data on human seasonal influenza viruses, including the activities of A (H1N1), A (H3N2), B/Victoria, and B/Yamagata. Data obtained through virology surveillance in temperate locations have demonstrated a consistent seasonal pattern with this seasonality being weaker in subtropical and tropical locations [1].

The WHO declared the COVID-19 pandemic on March 11, 2020. Accordingly, governments worldwide launched different levels of mitigation strategies. Subsequently, several northern-hemisphere countries showed a notable decrease in the acute respiratory tract infection or influenza-like illness (ILI) consultation rates at the end of the 2019/2020 season and during the 2020/2021 season compared with the rates in previous years [2-4]. In 2020, there were decreased influenza burdens and activity in southern-hemisphere countries, which varied among the countries [5]. In China, compared to the average levels during 2012-2019, the positive rate of influenza virus decreased during 2020 with changes of –87.6% [6]. Similar variations have been observed for other respiratory viruses in China and the United States [6,7].

Decreased influenza transmission dynamics during the 2019/2020 season mainly resulted from the public health and social measures implemented to mitigate the COVID-19 pandemic [8,9]. The reducing outpatient ILI consultations in cities of China were also caused by the mitigation measures [10]. Influenza activity and seasonal patterns were influenced by COVID-19, and the activity of these 2 diseases suppressed and competed with each other, showing a seesaw effect [11]. The study also found that population susceptibility increased in both China and the United States, which might lead to large, high-intensity influenza outbreaks in the 2022/2023 season [12]. Furthermore, the lack of exposure to influenza together with unpredictable antigenic changes may have reduced population immunity, which could lead to a more severe influenza season after the COVID-19 mitigation measures are lifted [13].

Prior to the COVID-19 pandemic, influenza virus subtypes and lineages were cocirculated and the predominant subtypes varied each year, while after the pandemic the B/Victoria lineage dominated in 2020/2021 in Chongqing, China [14]. Large changes occurred in influenza subtype distribution during the pandemic, including the sharp decrease in the global prevalence of B/Yamagata viruses and a temporal increased prevalence of B/Victoria virus in the Western Pacific Region [15]. The unexpected activities of these viruses were potential risks. Therefore, there is a need to elucidate variations in the epidemiology and transmission characteristics of the influenza virus since the COVID-19 pandemic [16]. Meanwhile, it is necessary to monitor and update knowledge on the circulation types and subtypes to prepare for future outbreaks [17]. Moreover, given the uncertainty regarding incoming influenza strains and the influence of COVID-19 prevention measures, it is important to perform surveillance of influenza activity and reevaluate the influence of COVID-19 measures on it. Using influenza surveillance data collected from the WHO, we aimed to describe the epidemiologic characteristics of influenza from 2012 to 2023 as well as to explore the correlation between influenza activity and public health and social measures.

## Methods

### Data Collection

**Figure 1:**
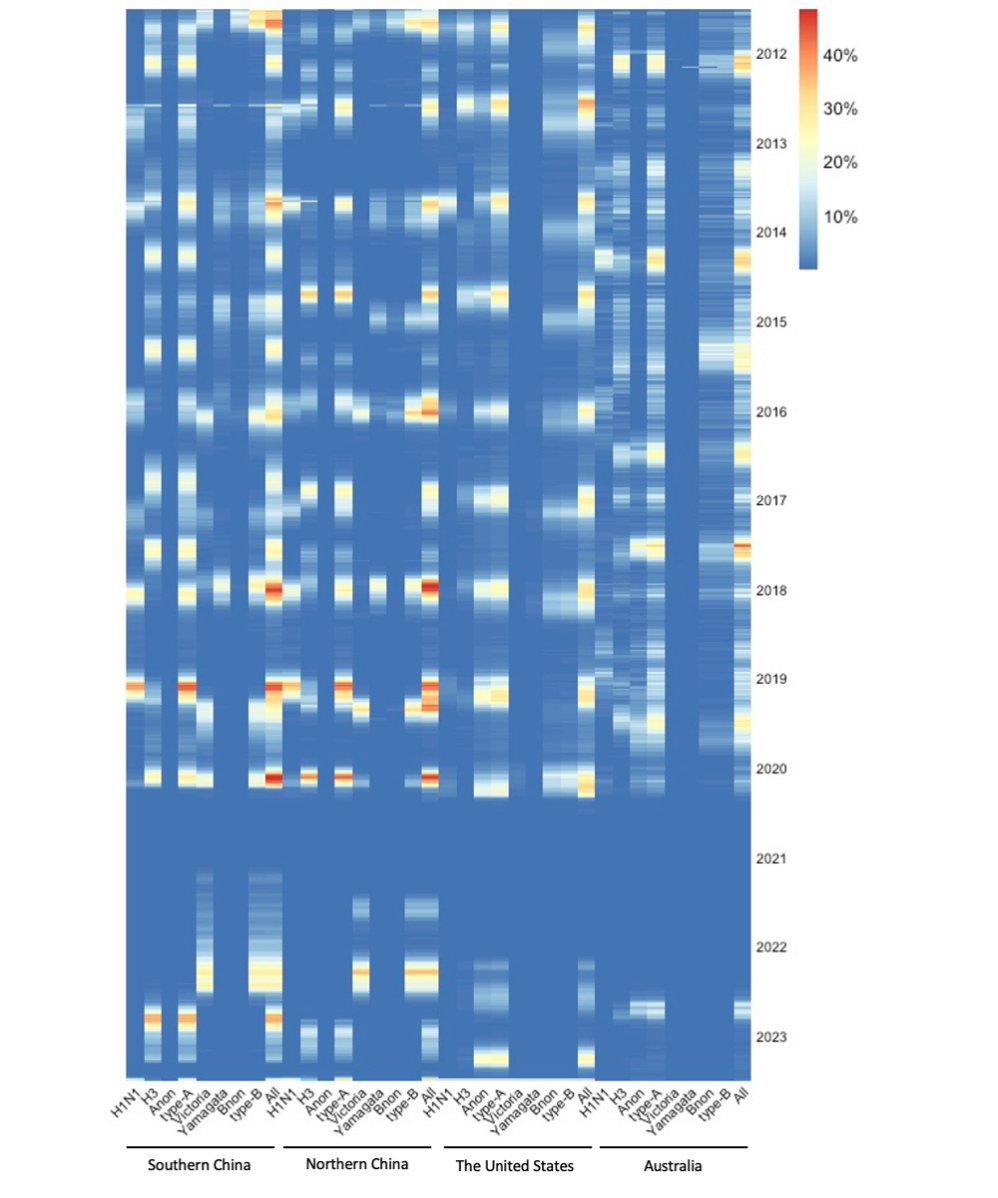
Seasonal influenza activity from 2012 to early 2023 with virus types or subtypes across different areas. Surveillance data are obtained from WHO FluNet and the Chinese National Influenza Center database.

**Figure 2:**
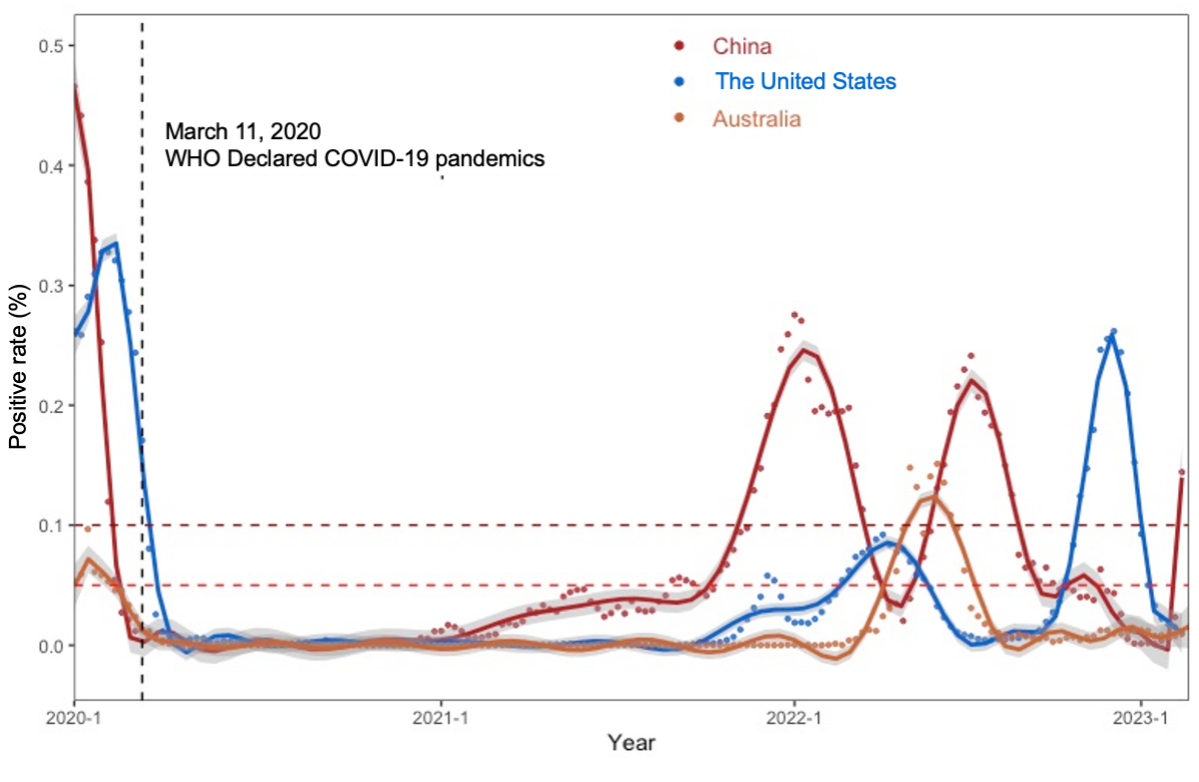
Influenza activity after the COVID-19 pandemic in China, the United States, and Australia. Surveillance data are obtained from WHO FluNet and the Chinese National Influenza Center database. The dark red and red dot lines indicate positivity rates of 10% and 5%, respectively.

**Figure 3:**
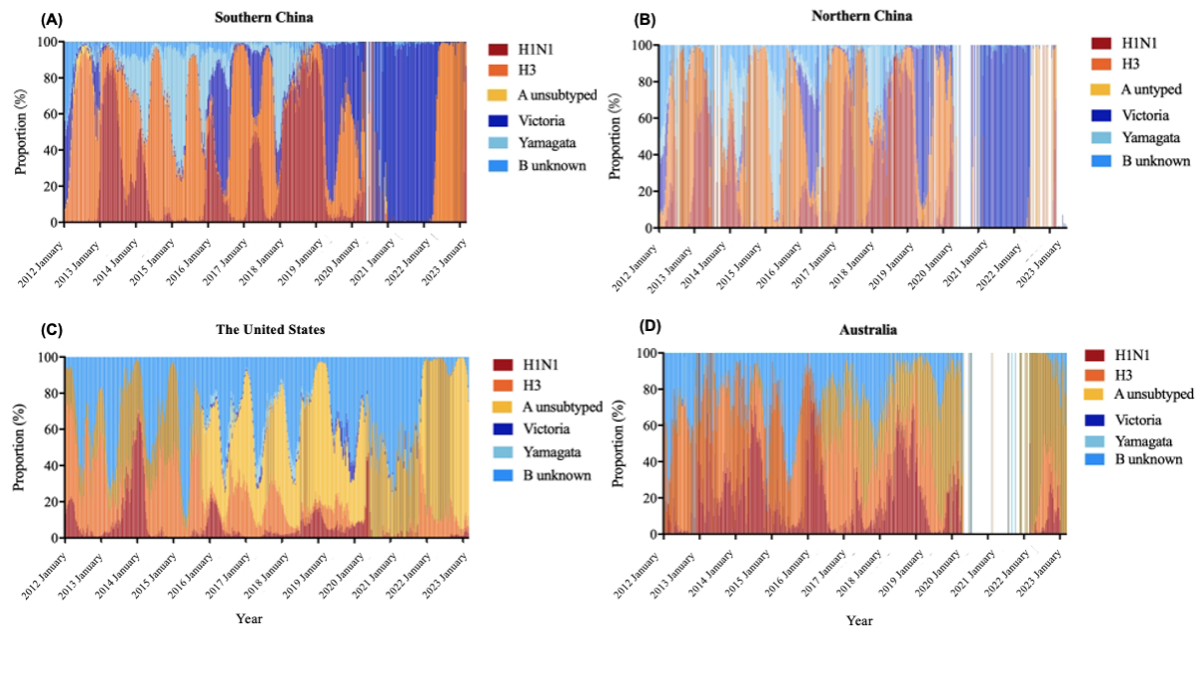
Influenza virus subtypes distribution from 2012 to early 2023 among different areas. (A) Southern China. (B) Northern China. (C) The United States. (D) Australia. Red indicates the presence of the H1N1 influenza virus. Orange indicates the H3 influenza virus. Yellow indicates the unsubtyped influenza A. Dark blue indicates the influenza virus of the Victoria lineage. Light blue indicates the influenza virus of the Yamagata lineage. Blue indicates an unknown influenza virus of type B. The white areas indicate no reported cases of influenza from Australia to the FluNet during the period from 2020 week 17 to 2022 week 6, and no reported cases to the Chinese National Influenza Center database from 2020 week 16 to 2020 week 53 in China.

We obtained influenza data from January 1, 2012, to February 5, 2023 (data download on February 17, 2023) for the countries and areas of the United States, and Australia from the publicly available platform WHO FluNet, which is remotely provided by the National Influenza Centers of the Global Influenza Surveillance and Response System (GISRS). We also obtained data for the southern and northern areas of China from the Chinese National Influenza Center database. We analyzed data differently by region in China because influenza epidemiologic features differ between the South and North [8]. Meanwhile, the 2 regions have different climatic characteristics. We chose these areas because they are members of WHO Collaborating Centers, meanwhile, the United States and Australia are also members of the WHO Essential Regulatory Laboratories within GISRS, and we believed that there would be accurate data reported to the GISRS from these areas. Meanwhile, China, the United States, and Australia were selected as countries representing each of the 3 WHO transmission zones, where each zone covers a geographical group of countries with different influenza transmission patterns [18]. China and the United States both include tropical, subtropical, and temperate zones, and they are part of the Eastern Asia influenza transmission zone, and the North America influenza transmission zone, respectively. Australia is mainly temperate and located in the Oceania, Melanesia, and Polynesia influenza transmission zones. The influenza activity from 2012 to 2023 is detailed in Figure 1, Table 1, and Multimedia Appendix 1. Then the influenza-positive rates among surveillance samples are further illustrated in Figure 2, and the proportion of influenza subtypes is detailed in Figure 3.

**Table 1:** Comparison of influenza activity features during 2017-2022 in China, the United States, and Australia.

Years		2021/2022	2020/2021	2019/2020	2018/2019	2017/2018
**Southern China^a^**						
	Influenza season duration (weeks)	22	0	12	31	39
	Influenza activity peak, n/N (%)	1471/5244 (28.1)	387/4565 (8.5)	2262/4676 (48.4)	4527/9935 (45.6)	3551/7615 (46.6)
	Influenza virus type A, n/N (%)	1/5244 (0)	0/4565 (0)	1300/4676 (27.8)	4503/9935 (45.3)	1915/7615 (25.2)
	Influenza virus type B, n/N (%)	1470/5244 (28)	387/4565 (8.5)	962/4676 (20.8)	24/9935 (0.2)	1636/7615 (21.5)
	Week of peak	Week 2, 2022	Week 39, 2021	Week 1, 2020	Week 3, 2019	Week 3, 2018
**Northern China^a^**						
	Influenza season duration (week)	17	0	10	25	18
	Influenza activity peak, n/N (%)	1738/4946 (35.1)	228/2605 (8.75)	2363/5031 (47)	2547/5681 (44.8)	2874/5931 (48.5)
	Influenza virus type A, n/N (%)	0/4946 (0)	0/2605 (0)	2175/5031 (43.2)	2513/5681 (44.2)	1500/5931 (25.3)
	Influenza virus type B, n/N (%)	1738/4946 (35.1)	228/2605 (8.75)	188/5031 (3.7)	34/5681 (0.6)	1374/5931 (23.2)
	Week of peak	Week 2, 2022	Week 21, 2021	Week 1, 2020	Week 3, 2019	Week 1, 2018
**United States^a^**						
	Influenza season duration (week)	0	0	17	19	21
	Influenza activity peak, n/N (%)	7682/83,426 (9.2)	72/26,843 (0.3)	25,412/77,514 (32.8)	17,178/58,082 (29.6)	22,933/75,195 (30.5)
	Influenza virus type A, n/N (%)	7652/83,426 (9.2)	38/26,843 (0.1)	15,461/77,514 (20)	16,660/58,082 (28.7)	18,948/75,195 (25.2)
	Influenza virus type B, n/N (%)	30/83,426 (0)	34/26,843 (0.1)	9951/77,514 (12.8)	518/58,082 (0.9)	3985/75,195 (5.3)
	Week of peak	Week 15, 2022	Week 41, 2020	Week 6, 2020	Week 9, 2019	Week 2, 2018
**Australia^b^**						
	Influenza season duration (week)	7	0	0	40	12
	Influenza activity peak, n/N (%)	1460/9662 (15.1)	1/1606 (0.1)	113/1170 (9.7)	912/3119 (29.2)	73/409 (17.9)
	Influenza virus type A, n/N (%)	1455/9662 (15.1)	1/1606 (0.1)	103/1170 (8.8)	844/3119 (27.1)	55/409 (13.5)
	Influenza virus type B, n/N (%)	5/9662 (0.1)	0/1606 (0)	10/1170 (0.9)	68/3119 (2.2)	18/409 (4.4)
	Week of peak	Week 24, 2022	Week 6, 2021	Week 4, 2020	Week 23, 2019	Week 7, 2018

^a^From week 40 of the previous year to week 39 of the next year.

^b^From week 45 of the previous year to week 44 of the next year.

**Figure 4:**
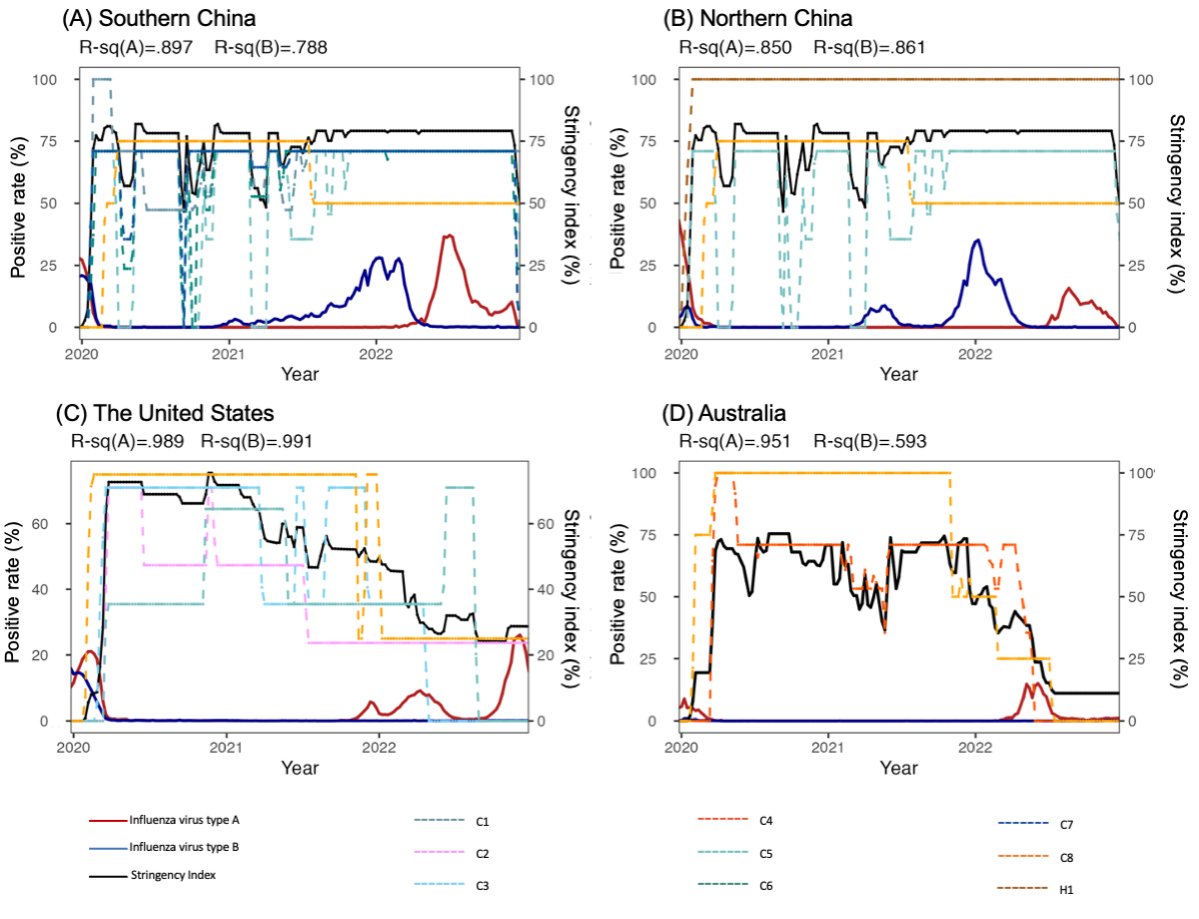
Correlation between the stringency index and the influenza positivity rate in different countries or areas since the COVID-19 pandemic. (A) Southern China. (B) Northern China. (C) The United States. (D) Australia. The dark line represents the stringency index. The red line represents the positivity rate of influenza type A, and the blue line represents the positivity rate of influenza type B. R-sq(A) represents the determination coefficient between influenza type A and the stringency index. In contrast, R-sq(B) represents the determination coefficient between influenza type B and the stringency index. Dashed lines indicate different management measures, including C1, C2, C3, C4, C5, C6, C7, C8, and H1. The measures that significantly affected influenza activity are displayed in the figure. C1: school closing; C2: workplace closing; C3: canceling public events; C4: restrictions on gathering size; C5: closing public transport; C6: stay-at-home requirements; C7: restrictions on internal movement; C8: restrictions on international travel; H1: public information campaign.

The stringency index (SI) is a composite index for measuring the strictness of “lockdown style” policies that primarily restrict people’s behaviors, and it is rescaled to a value from 0 to 100 (100=strictest). It was proposed by the Balavatnik School of Government, University of Oxford [19]. The SI is calculated using 9 metrics as follows: C1, school closing; C2, workplace closing; C3, cancel public events; C4, restrictions on gathering size; C5, close public transport; C6, “shelter-in-place” and home confinement orders; C7, restrictions on internal movements; C8, restrictions on international travel; and H1, public information campaign. Each index for all the 9 strategies was calculated and acquired from the publicly available platform proposed by the Balavatnik School of Government, University of Oxford [20]. This data was downloaded on September 27, 2023, and updated until December 31, 2022. Further analysis of the correlation between influenza activity and SI was detailed in Tables 2 and 3, Figure 4, and Multimedia Appendix 1.

**Table 2:** Influence of individual management measure on influenza virus A and B activity in China, the United States, and Australia during 2020-2022^a^.

Measures	Southern China		Northern China		United States		Australia	
	DE_A_^b^ (%, *P* value)	DE_B_ (%, *P* value)	DE_A_ (%, *P* value)	DE_B_ (%, *P* value)	DE_A_ (%, *P* value)	DE_B_ (%, *P* value)	DE_A_ (%, *P* value)	DE_B_ (%, *P* value)
C1^c^	65.8 (<.001)^d^	43.8 (<.001)^d^	77.4 (<.001)^d^	48.2 (<.001)^d^	88.5 (<.001)^d^	94 (<.001)^d^	78 (<.001)^d^	41.6 (.18)
C2^e^	51 (<.001)^d^	51.2 (<.001)^d^	70 (<.001)^d^	56.5 (<.001)^d^	80.9 (<.001)^d^	97.5 (<.001)^d^	88.5 (<.001)^d^	69.8 (.04)^d^
C3^f^	38.3 (<.001)^d^	34.6 (.01)^d^	59.9 (<.001)^d^	39.1 (<.001)^d^	91.1 (<.001)^d^	90.1 (<.001)^d^	84.6 (<.001)^d^	69.4 (.049)^d^
C4^g^	35.9 (<.001)^d^	36 (<.001)^d^	55.7 (<.001)^d^	40.5 (<.001)^d^	92 (<.001)^d^	91.2 (<.001)^d^	87.1 (<.001)^d^	67.9 (.02)^d^
C5^h^	47.5 (<.001)^d^	36.4 (.001)^d^	53.6 (<.001)^d^	44.7 (<.001)^d^	83.8 (<.001)^d^	94.8 (<.001)^d^	50.3 (<.001)^d^	73.8 (.06)
C6^i^	37.2 (<.001)^d^	37.2 (<.001)^d^	54.2 (<.001)^d^	54.2 (<.001)^d^	79.6 (<.001)^d^	94.9 (<.001)^d^	66 (<.001)^d^	43.1 (.19)
C7^j^	36.2 (<.001)^d^	37.6 (.004)^d^	51.9 (<.001)^d^	43.4 (<.001)^d^	54.1 (<.001)^d^	77.9 (.001)^d^	63.2 (<.001)^d^	62 (.04)^d^
C8^k^	80.7 (<.001)^d^	53.2 (<.001)^d^	76.6 (<.001)^d^	53.2 (<.001)^d^	65.2 (<.001)^d^	77.7 (<.001)^d^	84.3 (<.001)^d^	46.8 (.03)^d^
H1^l^	37.8 (<.001)^d^	34.7 (.04)^d^	59.6 (<.001)^d^	39.4 (<.001)^d^	53.9 (<.001)^d^	97.6 (<.001)^d^	40.3 (<.001)^d^	45 (.02)^d^

^a^A implies the activity of influenza virus type A and B implies the activity of influenza virus type B.

^b^DE: deviance explained.

^c^C1: school closing.

^d^Significant at *P*<.001.

^e^C2: workplace closing.

^f^C3: cancel public events.

^g^C4: restrictions on gathering size.

^h^C5: close public transport.

^i^C6: “shelter-in-place” and home confinement orders.

^j^C7: restrictions on internal movements.

^k^C8: restrictions on international travel.

^k^H1: public information campaign.

**Table 3:** Influence of all the 9 management measures combined on influenza virus A and B activity in China, the United States, and Australia during 2020-2022

Measures		Southern China							Northern China							United States							Australia				
		Deviance explained		*P* value (A)		*P* value (B)		Deviance explained			*P* value (A)		*P* value (B)		Deviance explained			*P* value (A)		*P* value (B)		Deviance explained			*P* value (A)		*P* value (B)
		87.4% (A), 77.6% (B)						83.4% (A), 81.4% (B)							98.6% (A), 99.1% (B)							95.8% (A), 72.7% (B)					
C1^b^			.72		<.001^c^					.003^c^		.04^c^					.90		.35					.44		—^d^	
C2^e^			.62		.34					.35		.97					.04^c^		.46					.06		.73	
C3^f^			.13		.48					.11		.16					.03^c^		.96					.31		.59	
C4^g^			.15		.15					.22		.08					.22		.75					<.001^c^		.62	
C5^h^			.002^c^		<.001^c^					.02^c^		<.001^c^					.01^c^		.76					.13		—	
C6^i^			.13		.001^c^					.36		.42					.14		.25					.26		—	
C7^j^			.21		.001^c^					.16		.07					.61		.99					.43		.67	
C8^k^			<.001^c^		<.001^c^					.06		.001^c^					.30		.02^c^					.04^c^		.80	
H1^l^			.16		.87					.36		.049					.004^c^		<.001^c^					.30		.49	

^a^A implies the activity of influenza virus type A and B implies the activity of influenza virus type B.

^b^C1**:** school closing**.**

^c^Significant at *P*<.05.

^d^Not applicable.

^e^C2: workplace closing.

^f^C3: cancel public events.

^g^C4: restrictions on gathering size.

^h^C5: close public transport.

^i^C6: “shelter-in-place” and home confinement orders.

^j^C7: restrictions on internal movements.

^k^C8: restrictions on international travel.

^l^H1: public information campaign.

### Related Definitions

All of these 3 areas test for influenza virus through ILI cases, and different countries followed their own definition for the diagnosis of ILI. An ILI case in China is defined as a fever (temperature ≥38 ℃) with a cough or sore throat [21]. ILI in the United States is defined as a fever (temperature of 100 °F [37.8 °C] or greater) and a cough or a sore throat [22]. The Communicable Diseases Network Australia guidelines define ILI as a triad of fever ≥38 °C, respiratory symptoms, and systemic symptoms [23]. The WHO case definition for ILI is an acute respiratory tract infection with temperature ≥38 ℃ and cough, with onset within the last 10 days [23].

The number of specimens that tested positive for influenza virus (with confirmation of the type and subtype) is presented as a percentage of the total number of tested specimens. An influenza positivity rate of ≥10% and <10% for ≥2 consecutive weeks indicates the start and end of seasonal influenza activity, respectively. Weeks were defined using the International Organization for Standardization 8601 standard weeks. Each season was analyzed from week 40 of the previous year to week 39 of the next year in China and the United States, and for Australia, it was from the 45th week of the previous year to the 44th week of the next year.

### Statistical Analysis

Heat maps were applied to depict the influenza virus subtypes epidemiologic characteristics from 2012 to early 2023 (Figure 1). The influenza virus subtypes proportion was illustrated from 2012 to early 2023, and graphs were generated by Prime 8 (version 8.3.1; GraphPad Software, LLC; Figure 3). In the current analysis, we used the SI as an index for the evaluation of public health and social measures (Tables 2 and 3, Figure 4, and Multimedia Appendix 1). The value of the index is the average of 9 subindices about the individual policy indicators, each taking a value from 0 to 100. The Balavatnik School of Government provided daily SI, and the daily SI data were converted to weekly averages to be consistent with the ILI data, detailed in Multimedia Appendix 1. The general additive model was used to assess the correlation between the SIs (defined as an independent variable, X_i_) and the influenza-positive rate (defined as the dependent variable, *Y_t_*). The deviance explained was calculated to estimate the contribution value of the independent variable to the dependent variable, and the determination coefficient (*R*^2^) was calculated to assess the goodness of fit of the models (Multimedia Appendix 1).

Statistical analyses were conducted using R (version 4.2.3; The R Foundation). Statistical significance was set at *P*<.05.

### Ethical Considerations

This study was conducted with data from publicly available platforms, where the data were published. The research involves the use of collections of information or data from which all personal identifiers have been removed before being received by the researchers. Additionally, according to the ethical requirement from China (article 32), the United States (page 4), and Australia (section 5, article 5.1.17), the research using these data may be eligible for a grant of exemption from ethics review [24-26].

## Results

### Characteristics Regarding Seasonal Influenza Activity From 2012 to Early 2023

We used over 200,000 influenza surveillance data in the current analysis. In mainland China, the United States, and Australia, the influenza activity showed obvious seasonal peaks previous to the COVID-19 pandemic. Figure 1 shows the distribution of all influenza genotypes from 2012 to 2023 (Figure 1). The influenza epidemiologic features in these 4 regions differed before and during the pandemic, and the new epidemiologic features are also different in each area. Since the COVID-19 pandemic, there has been a gradual decrease in the influenza positivity rate, which was <10% in China and Australia by the time WHO declared COVID-19 a pandemic (Figure 2). The influenza positivity rate remained <5% during the 2020/2021 season in all 4 regions. Influenza activity remained low in the United States, and Australia during the 2021/2022 season, with peak positivity rates of 9.2% (mainly influenza A), 7682 of 83,426 surveillance samples, in week 15, 2022, and 15.1% (mainly influenza A), 1460 of 9662 surveillance samples, in week 24, 2022, respectively. During the 2021/2022 season, influenza activity significantly increased compared with the previous year in southern and northern China, with peak positivity rates of 28.1% (mainly influenza B), 1471 of 5244 surveillance samples, and 35.1% (mainly influenza B), 1738 of 4946 surveillance samples, in the second week of 2022, respectively, and with 2 seasonal influenza activity peaks during winter and summer in northern instead of southern China (Figure 2 and Table 1).

For China, the influenza season durations were significantly shorter during the 2019/2020, and 2020/2021 seasons, however, the durations tended to be similar to the prepandemic during the 2021/2022 season (22 weeks in the south and 17 weeks in the north). The influenza season durations were significantly shorter during the 2020/2021 (0 weeks) and 2021/2022 seasons (0 weeks) in the United States, and during the 2019/2020 (0 weeks), 2020/2021 (0 weeks), and 2021/2022 seasons (7 weeks) in Australia (Table 1). For China and Australia, compared to the 2020/2021 season, the influenza season duration was much longer, and the activity peaks were higher during the 2021/2022 season (Table 1). For the United States, influenza activity increased with a positivity rate of over 10% at the end of 2022 (Figure 2).

### Variations in the Features of the Influenza Virus Since the COVID-19 Pandemic

Figure 3 details the influenza virus types and subtypes. During the last 8 prepandemic years, type A was the dominant influenza virus genotype for most of the time in all 3 areas. The dominant influenza virus genotype after the COVID-19 pandemic gradually became type B (mainly Victoria) in China during the winter of 2021/2022 (proportion >98%, 24,541/24,908 in the South and 20,543/20,634 in the North). Then in April 2022, type B/Victoria was decreased. However, type A (H3) became the dominant subtype (proportion >99%) in southern China (18,667/18,822) first and in northern China (3268/3278) later. Contrastingly, the dominant influenza virus genotype during the 2021/2022 season was type A in the United States and Australia (proportion >90%, 153,735/155,565 and 8752/8769, respectively) for the 2 countries. In all these 4 areas, influenza virus type B/Yamagata has not been detected since the COVID-19 pandemic.

### Correlation of Public Health and Social Measures for COVID-19 With Seasonal Influenza Activity

Seasonal influenza activity was impacted by the COVID-19 pandemic. We analyzed the correlation between the SI and the influenza positivity rate (Tables 2 and 3, and Figure 4). All 9 management measures were individually evaluated and detailed in Table 2. Most of these measures significantly affected the positive rate of influenza types A and B. However, for influenza B in Australia, measures C1 (school closing), C5 (closing public transport), and C6 (shelter-in-place and home confinement orders) affected the positive rate without significant differences (Table 2).

We further analyzed the combined effect of all measures on influenza-positive rates (Table 3 and Figure 4). When combined, measures C1 (school closing), C5 (closing public transport), C6 (shelter-in-place and home confinement orders), C7 (restrictions on internal movements), and C8 (restrictions on international travel) significantly affected influenza A or B activity (*P*<.05) in southern China. In northern China, C1 and C5 significantly affected influenza (both A and B) activity (*P*<.05), and C8 significantly affected influenza B activity (*P*<.05). The deviance explained value for influenza A was 87.4% and for influenza B was 77.6% in southern China, while for influenza A and B in northern China, it was 83.4% and 81.4%, respectively. In the United States, C2 (workplace closing), C3 (cancel public events), C5, and H1 (public information campaign) significantly affected the influenza A positive rate, and C8 and H1 affected the positive rate of influenza B. The correlation between the SI and influenza activity was relatively stronger, with deviance-explained values of 98.6% for influenza A and 99.1% for influenza B in the United States (Table 3). In Australia, C5 and C8 affected influenza A activity significantly (*P*<.05). As the positive rate of influenza B was extremely low from 2020 to 2022, no significant effects were found on the influenza B activity. The correlation between the SI and the influenza positivity rate in Australia was 95.8% for influenza type A and 72.7% for influenza type B. However, despite the high level of the SI in China, influenza activity increased in 2021 compared to 2020.

## Discussion

### Principal Results

We analyzed influenza surveillance data from China, the United States, and Australia to describe influenza activity within 10 years before and after the COVID-19 pandemic. The implementation of public health and social measures in China and the United States contributed to decreased influenza activity during the 2019/2021 season [7,8]. However, we observed a gradual increase in influenza activity starting from the beginning of 2021 reaching 28.1% (1471/5244) in southern China and 35.1% (1738/4946) in northern China by the second week of 2022. Contrastingly, influenza activity in the United States, and Australia during the 2021/2022 season remained low, even though it was slightly higher than during the 2020/2021 season (Figure 2 and Table 1). However, by the end of 2022 and early 2023, influenza activity increased in the United States, with a positivity rate of 26.2% in the 49th week of 2022. Notably, the dominant influenza virus genotype since the COVID-19 pandemic in China, especially during the 2021/2022 season, was type B/Victoria, which then transformed to type A (H3) in April 2022. The dominant virus was type A in the United States and Australia during 2022 and early 2023. Postpandemic variations in influenza activity were correlated with the public health and social measures for COVID-19. However, it is important to consider that there may be other factors affecting influenza activity and emerging as new epidemiological features.

In Canada, surveillance data obtained during the 2021/2022 season revealed persistent sporadic influenza activity, with the predominant virus subtype being A (H3N2) [27]. Similarly, type A (H3) was predominant in the United States, with laboratory-confirmed influenza showing a low positivity rate during the 2021/2022 season. However, the positivity rate increased at the end of 2022 and then decreased at the beginning of 2023. In Australia, type A (H3) was dominant in May and June 2022, and then the influenza positivity decreased and maintained a low level until 2023. China showed unique changes in the dominant influenza virus type, which was type B/Victoria during the 2021/2022 season. Another study found that before the COVID-19 outbreak, influenza A viruses dominated in China, whereas influenza B/Victoria viruses dominated during the 2020/2021 season [15]. Our results underscored that type B/Victoria dominated in China until April 2022, with a positivity rate of over 25% in both southern and northern regions during the 2021/2022 winter. During the influenza season, type B usually results in mild symptoms and less frequent epidemics and has never caused pandemics in humans [28]. The epidemiological characteristics of influenza during the 2021/2022 season in China, specifically before January 2022, included single-type spreading and a relatively higher activity peak compared to the previous year. These characteristics could be attributed to restricted inbound travels and within-country transmission, which was also the dominant transmission mechanism for COVID-19 in China [29]. As results displayed in our study, in China during 2020-2021, the SI remained at a high level. However, the restrictions on population internal movements were fluctuant, and sometimes even measures such as “restrictions on internal movements” or “close public transport” were canceled (Figure S1 in Multimedia Appendix 1). This led to the emergence of influenza cases in 2021, albeit at a low level. Compared to domestic movement mitigation, the mitigation of international travel had a larger effect [30]. The measure of restrictions on international travel significantly affected the activities of influenza in China, and the positive rate of influenza (B/Victoria) increased by over 10% after the relaxation of international travel in 2021 (Figure 4 and Table 3). Influenza type A (H3N2) first emerged in southwestern China in early 2022, extensively spreading in southern China, and finally reaching northern China in July 2022. It may have entered China from Southeast Asia.

Currently, promoting vaccination coverage remains the primary strategy for controlling influenza [31]. The COVID-19 pandemic increased the likelihood of receiving an influenza vaccine in the United States [32], England [13], and Australia [33]. However, in China, the influenza vaccination rate remains low; moreover, parental acceptance of childhood vaccination is still low (29% in northwestern China) [34]. This could explain the relatively higher influenza activity in China compared with the United States, and Australia.

The low influenza activity during the 2020/2021 season is mainly attributable to public health and social measures [8]. A modeling study predicted that influenza activity would return to prepandemic levels during the 2021/2022 season after easing the current mitigation measures for COVID-19 [35]. Even though significant correlations between the SI and influenza positivity rates were found in Australia and the United States, these correlations were mainly driven by the early phase of the pandemic and fluctuations in mitigation measures during 2022. Although we observed a relatively higher influenza activity peak during the 2021/2022 season in China, the local mitigation measures were still strictly implemented, as indicated by the SI. The potential explanation might be that the SI was a composite index, and the high number of SIs for China might be driven by international travel controls instead of restrictions on internal movements (Figure S1 in Multimedia Appendix 1). However, there are differences in the effectiveness of various mitigation measures in reducing virus transmission. For example, a previous study showed that mask-wearing was more effective than mitigating travel or movement, while interventions targeting international travel were more effective than those targeting domestic travel [30]. Implementing optimized strategies with composite measures would help in quickly responding to unpredictable respiratory infectious diseases. Furthermore, it is necessary to evaluate the effectiveness of different measures, which can help establish optimized strategies in the future.

There are immunopathological similarities between the influenza virus and SARS-CoV-2 [36]. Additionally, influenza vaccination is associated with reduced susceptibility to or severity of COVID-19 [37]. Therefore, the observed correlations in this study might not be limited to the influence of public health and social measures. Multi-pathogen mutual interference might also play an important role in current influenza activities. During the current unstable phase, it is important to monitor influenza and other respiratory pathogens’ activities simultaneously. Rapid integration of multi-pathogen testing and surveillance would help prevent emerging pathogens from evolving into another pandemic [38]. Currently, it is crucial and timely to track the activity of influenza and re-evaluate the effect of COVID-19 mitigation measures, because as the pandemic progresses and after, more factors will emerge that affect influenza activity. Meanwhile, it is also essential to strictly surveil the seasonal influenza activity and the variation of pathogens because the COVID-19 pandemic influenced the features of seasonal activities.

### Limitations

This study has several limitations. First, the evaluation of the effect of other measures, such as mask-wearing, was not supported by the currently available data, despite their reported significant impact on influenza activities. Second, we did not consider climate and environmental factors. In the early stage of the COVID-19 pandemic, public health and social measures had the strongest influence on influenza activity [6]. However, temperature, wind speed, and a particulate matter level of 2.5 also influence the transmission of some respiratory infectious diseases [39].

### Conclusions

This study provides an updated report on influenza activity in 4 areas from 2012 to early 2023, as well as the impact of public health and social measures for COVID-19 on influenza activity. Our findings indicate an increase in influenza activity in these 3 countries. While there were correlations between mitigation measures for COVID-19 and influenza activity, the main driving force was the early stage of the pandemic. The role of mitigation management in influencing influenza activity varied across different stages of the pandemic and among different countries. It is crucial to maintain vigilance to prevent influenza and other respiratory pathogens from causing epidemics. Further studies are warranted to investigate the reasons behind the variations in dominant virus subtypes across regions and to assess the influence of individual mitigation measures, climate factors, and the activity of other pathogens on influenza.

## Appendix

Multimedia Appendix 1 Material, tables, and figures for the methods and results.

## Abbreviations

GISRS: Global Influenza Surveillance and Response System
ILI: influenza-like illness
SI: stringency index
WHO: World Health Organization

## Data Availability

Data on influenza activity are available on the WHO FluNet and data on the SI are available on the website of the Balavatnik School of Government, the University of Oxford. Processed datasets used in this analysis are available from the corresponding author upon reasonable request.
