# Supplementary material for: Changes in the Epidemiological Features of Influenza After the COVID-19 Pandemic in China, the United States, and Australia: Updated Surveillance Data for Influenza Activity
Source: Interact J Med Res. 2024 Oct 9;13:e47370. doi: 10.2196/47370 (PMC11499725; doi:10.2196/47370)
Supplement: Multimedia Appendix 1 [file ijmr_v13i1e47370_app1.docx]

**Methods and Results**

**Statistical Analysis**

The stringency index (SI) was used as an index for evaluation of the public health and social measures. The value of SI is the average of nine sub-indices about the individual policy indicators. The calculation of each indicator was conducted according to the Equation below:

$I_{j}\text{=100(}C_{j}\frac{\text{1-w}}{N_{j}}+wG_{j})$ (1)

*C_j_* is the ordinal value of the corresponding indicator; *N_j_* is the maximum value of the indicators; *w* is the weight for a policy of general scope is defined in relation to the number of ordinal points of the eight indicators that have the targeted/general flags, that is ≈ 0.29[1]. The description of all the indicators was detailed in table below.

**Table S1.** Description of each indicator.

| **ID** | **Name** | **Coding** |
| --- | --- | --- |
| C1 | school closing | 0 - No measures  1 - recommend closing  2 - Require closing (only some levels or categories, eg just high school, or just public schools)  3 - Require closing all levels  No data - blank  0 – Targeted   1. General   No data - blank |
| C2 | workplace closing | 0 - No measures  1 - recommend closing (or work from home)  2 - require closing (or work from home) for some sectors or categories of workers  3 - require closing (or work from home) all-but- essential workplaces (eg grocery stores, doctors) No data - blank  0 – Targeted   1. General   No data - blank |
| C3 | cancel public events | 1. No measures 2. Recommend cancelling   2 - Require cancelling  No data - blank  0 – Targeted  1- General  No data - blank |
| C4 | restrictions on gathering size | 0 - No restrictions  1 - Restrictions on very large gatherings (the limit is above 1000 people)  2 - Restrictions on gatherings between 100-1000 people  3 - Restrictions on gatherings between 10-100 people  4 - Restrictions on gatherings of less than 10 people  No data - blank  0 – Targeted  1 – General  No data - blank |
| C5 | close public transport | 0 - No measures  1 - Recommend closing (or significantly reduce volume/route/means of transport available)  2 - Require closing (or prohibit most citizens from using it)  0 - Targeted  1 - General  No data - blank |
| C6 | “shelter-in-place” and home confinement orders | 0 - No measures  1 - recommend not leaving house  2 - require not leaving house with exceptions for daily exercise, grocery shopping, and ‘essential’ trips  3 - Require not leaving house with minimal exceptions (e.g. allowed to leave only once every few days, or only one person can leave at a time, etc.)  No data - blank  0 – Targeted  1- General  No data – blank |
| C7 | restrictions on internal movements | 0 - No measures  1 - Recommend closing (or significantly reduce volume/route/means of transport) 2 - Require closing (or prohibit most people from using it)  0 – Targeted  1- General  No data - blank |
| C8 | restrictions on international travel | 0 - No measures  1 – Screening  2 - Quarantine arrivals from high-risk regions  3 - Ban on high-risk regions  4 - Total border closure  No data - blank |
| H1 | public information campaign | 0 -No COVID-19 public information campaign  1 - public officials urging caution about COVID-19  2 - coordinated public information campaign (e.g. across traditional and social media)  No data - blank  0 – Targeted  1- General  No data - blank |

The general additive model (GAM) was used to assess the correlation between the stringency indexes (defined as independent variable, X_i_) and the influenza positive rate (defined as dependent variable, *Y_t_*).To evaluate the influence of each indicator, the analysis included one type of indicator each time. The equation was used as below:

$log[ E(Y_{i})]=\alpha+\beta_{i}\left[ s\left( X_{i} , df \right) \right]+s(W_{i},df)$ (2)

Indicators with significant effect on influenza positive rate in the equation (2) were included in another model to evaluate the influence of all the indicators. The equation was used as below:

$log[E( Y_{t})]=\alpha+ \sum\beta_{i} s\left( X_{i},df \right)+s(W_{i},df)$ (3)

Yi is the positive rate of influenza virus among ILI in a given week i; E(Yi) is the expected weekly influenza positive rate in a given week i, and the link function is log(); Xi is the individual policy indicator, and$\beta_{i}$ is its coefficient; Wi is a time series of week numbers (1, 2…, 52); s() smooth term; $\alpha$ is the intercept.

**Results**

**Table S2.** Comparison of influenza activity features during 2012-2017 among in China, the USA, and Australia.

| **Years** | **2016/2017** | **2015/2016** | **2014/2015** | **2013/2014** | **2012/2013** |
| --- | --- | --- | --- | --- | --- |
| **Southern China^a^** |  | | | | |
| Influenza season duration | 31 weeks | 20 weeks | 29 weeks | 34 weeks | 32 weeks |
| Influenza activity peak | 26.7%(1511/5662) | 32.2%(2020/6276) | 26.2%(1486/5664) | 38.4%(2362/6144) | 27.0%(1166/4316) |
| Influenza virus type A | 25.8%(1461/5662) | 21.8%(655/6276) | 24.4%(1384/5664) | 27.2%(1671/6144) | 19.0%(818/4316) |
| Influenza virus type B | 0.9%(50/5662) | 10.4%(1365/6276) | 1.8%(102/5664) | 11.3%(691/6144) | 8.1%(348/4316) |
| Week of peak | 2017W34 | 2016W12 | 2015W31 | 2014W2 | 2013W1 |
| **Northern China^a^** |  | | | | |
| Influenza season duration | 19 weeks | 18 weeks | 21 weeks | 16 weeks | 11 weeks |
| Influenza activity peak | 25.7%(1421/5529) | 42.0%(2489/5932) | 34.8%(2057/5913) | 34.3%(2097/6120) | 28.1%(1065/3794) |
| Influenza virus type A | 25.5%(1409/5529) | 33.5%(505/5932) | 34.5%(2037/5913) | 25.8%(1576/6120) | 27.9%(1060/3794) |
| Influenza virus type B | 0.2%(12/5529) | 8.5%(1984/5932) | 0.3%(20/5913) | 8.5%(521/6120) | 0.1%(5/3794) |
| Week of peak | 2016W52 | 2016W11 | 2014W52 | 2014W3 | 2013W2 |
| **The USA^a^** |  | | | | |
| Influenza season duration | 18 weeks | 14 weeks | 21 weeks | 24 weeks | 23 weeks |
| Influenza activity peak | 28.2%(14783/52446) | 28.6%(11971/41876) | 32.4%(13747/42466) | 30.6%(4478/14631) | 38.2%(6445/16881) |
| Influenza virus type A | 21.4%(11226/52446) | 22.0%(9212/41876) | 31.5%(13355/42466) | 30.0%(4391/14631) | 31.3%(5283/16881) |
| Influenza virus type B | 6.8%(3557/52446) | 6.6%(2759/41876) | 0.9%(392/42466) | 0.6%(87/14631) | 6.9%(1162/16881) |
| Week of peak | 2017W8 | 2016W10 | 2014W52 | 2013W52 | 2012W52 |
| **Australia^b^** |  | | | | |
| Influenza season duration | 21 weeks | 16 weeks | 31 weeks | 34 weeks | 16 weeks |
| Influenza activity peak | 42.9%(817/1905) | 28.8%(569/2025) | 28.8%(251/875) | 34.5%(277/804) | 22.1%(159/720) |
| Influenza virus type A | 32.0%(610/1905) | 24.1%(488/2025) | 13.0%(114/875) | 33.3%(268/804) | 17.6%(127/720) |
| Influenza virus type B | 10.9%(207/1905) | 4.0%(81/2025) | 15.7%(137/875) | 1.1%(9/804) | 4.4%(32/720) |
| Week of peak | 2017W32 | 2016W33 | 2015W34 | 2014W32 | 2013W35 |

Note: ^a^ From week 40 of the previous year to week 39 of the next year, ^b^ From week 45 of the previous year to week 44 of the next year.

Influenza activity features during 2012-2017 was detailed in Table S2. The influenza activity maintained a relatively stable epidemic pattern from 2012 to 2017


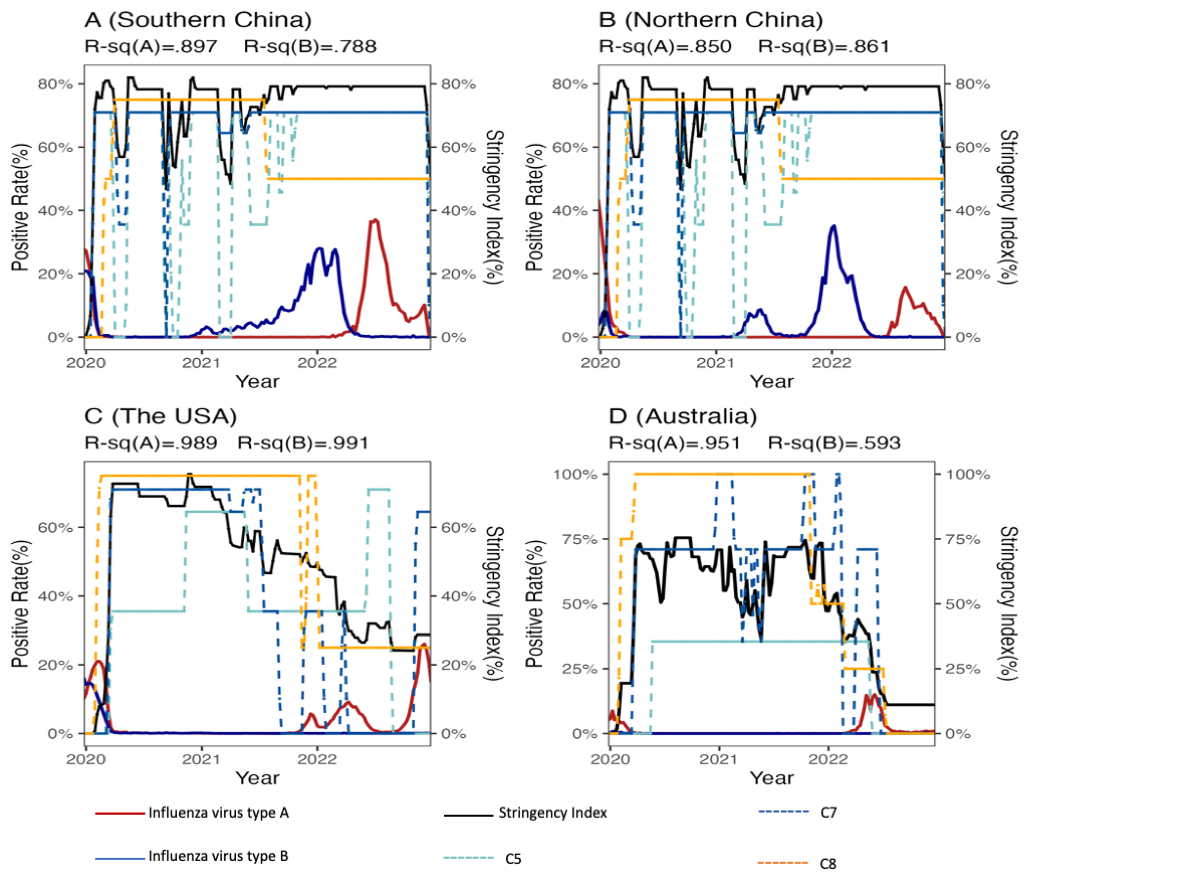


**Figure S1.** Correlation between the population movement and the influenza positivity rate in different countries/areas since the COVID-19 pandemic. (A) Southern China. (B) Northern China. (C) The USA. (D) Australia. The dark line represents the stringency index, while the red line represents the positivity rate of influenza type A, and the blue line represents the positivity rate of influenza type B. R-sq(A) represents the determination coefficient between influenza type A and the stringency index, while R-sq(B) represents the determination coefficient between influenza type B and the stringency index. Dashed lines indicate different management measures, including C5 (closing public transport), C7 (restrictions on internal movement), and C8 (restrictions on international travel).

This figure illustrated the influence of internal and international travel restrictions on influenza activities.

Reference

1. Tracker OC-GR. Calculation and presentation of the Stringency Index 4.0. 2020; Available from: https://www.bsg.ox.ac.uk/sites/default/files/Calculation%20and%20presentation%20of%20the%20Stringency%20Index.pdf.
